# The first description of dermal armour in snakes

**DOI:** 10.1038/s41598-023-33244-6

**Published:** 2023-04-19

**Authors:** Petra Frýdlová, Veronika Janovská, Jana Mrzílková, Milada Halašková, Markéta Riegerová, Jan Dudák, Veronika Tymlová, Jan Žemlička, Petr Zach, Daniel Frynta

**Affiliations:** 1 Department of Zoology, Faculty of Science, Charles University, 128 43 Prague, Czech Republic; 2 Department of Anatomy, Third Faculty of Medicine, Charles University, 100 00 Prague, Czech Republic; 3 Department of Histology and Embryology, Third Faculty of Medicine, Charles University, 100 00 Prague, Czech Republic; 4 Institute of Experimental and Applied Physics, Czech Technical University in Prague, 110 00 Prague, Czech Republic

**Keywords:** Evolution, Zoology, Ecology

## Abstract

Osteoderms, also called dermal armour, often play a role in predator defence. The presence of osteoderms is highly irregularly distributed across the squamate phylogeny and they have not been found in snakes. In this study, we searched for candidate snake species that would benefit from such armour to protect their body, focusing primarily on fossorial species with defensive tail displays. We examined the tail morphology of 27 snake species from different families using micro-computed tomography (µCT) and micro- radiography. We discovered dermal armour in four species of sand boas (Erycidae) that also feature enlarged and highly modified caudal vertebrae. This is the first description of dermal armour in snakes. Ancestral state reconstructions revealed that osteoderms likely evolved once or multiple times in Erycidae. We have not found osteoderms in any other examined snake species. Nevertheless, similar structures are known from unrelated squamate clades, such as gerrhosaurids and geckos. This supports the idea of underlying deep developmental homology. We propose the hypothesis that osteoderms protect sand boas like the “brigandine armour” of medieval warriors. We interpret it as another component of the sand boas' rich defence strategy.

## Introduction

There is a wide variety of integumentary elements in tetrapods, including osteoderms, dermal scales, and lamina calcarea^1^. While dermal scales and lamina calcarea are rather unique and typical of gymnophionans^2–4^ and some frogs^5–7^, osteoderms are common to representatives of most major taxonomic lineages^1^. An osteoderm is a structural category of mineralised organs incorporated into the dermis^8,9^. The literature is replete with routinely used synonyms such as dermal ossification, dermal plate, dermal armour, osteoscute, and scute^10–14^. In most tetrapods, osteoderms are not present until adulthood^1,15^ and remarkably little is known about their development during ontogeny^16,17^.

Osteoderms have been demonstrated in stem tetrapods^18,19^, amphibians^20^, lepidosaurs^21,22^, archosaurs^11,23,24^, and some synapsids^25–27^. Although dermal ossification is taxonomically widespread, its specific phylogenetic distribution across lineages is highly irregular. Attempts to reconstruct the evolution of dermal ossification yielded conflicting scenarios. The idea of convergent evolution of osteoderms, which has been put forward in amniotes^28^, has recently been replaced by the hypothesis of deep homology. It states that tetrapods share a plesiomorphic latent ability (genetic, cellular, developmental, and structural) to form such structures^11,25,29^.

Osteoderms have been reported in representatives of most major lepidosaur lineages^29–31^, e.g., in Sphenodontidae^32^, Gekkota^22,33,34^, Scincoidea^35^, Lacertoidea^36^, Anguimorpha^21,37–39^, and Iguania^40^ sensu^41^. In some families, osteoderms are widespread, e.g., in cordylids, gerrhosaurids, scincids, and anguids^42,43^. On the other hand, in Iguania or Gekkotans, only a few species among hundreds show well-developed osteoderms^29^. They have been repeatedly reported as absent in all snakes, amphisbaenians, dibamids, teiids and gymnophthalmids^1,29^. For the detailed mapping of the presence/absence of osteoderms to a phylogenetic tree of squamates see the review of Williams and her colleagues^29^.

Dermal ossification in squamate reptiles has been a hot topic in the past decade as the development and availability of micro-computed tomography (µCT) equipment has made it easier to study this phenomenon^14^. The non-invasiveness of µCT makes it easy to examine hundreds of museum specimens and fossils, as well as test specific hypotheses^44–48^. Furthermore, in vivo protocols^13^ allow for longitudinal studies with repeated measurements on living small animals^49^. With extending knowledge about the presence of osteoderms in squamates, we can hypothesise about their function. As osteoderms are mostly located on the head and dorsal surface of the body^30,42^, the most widely accepted hypothesis about the function of dermal armour is that it serves for protection^22,40^. Nevertheless, osteoderms may contribute to a variety of other functions, such as locomotion^50^, thermoregulation^44,51^, and calcium storage^52^.

**Figure 1:**
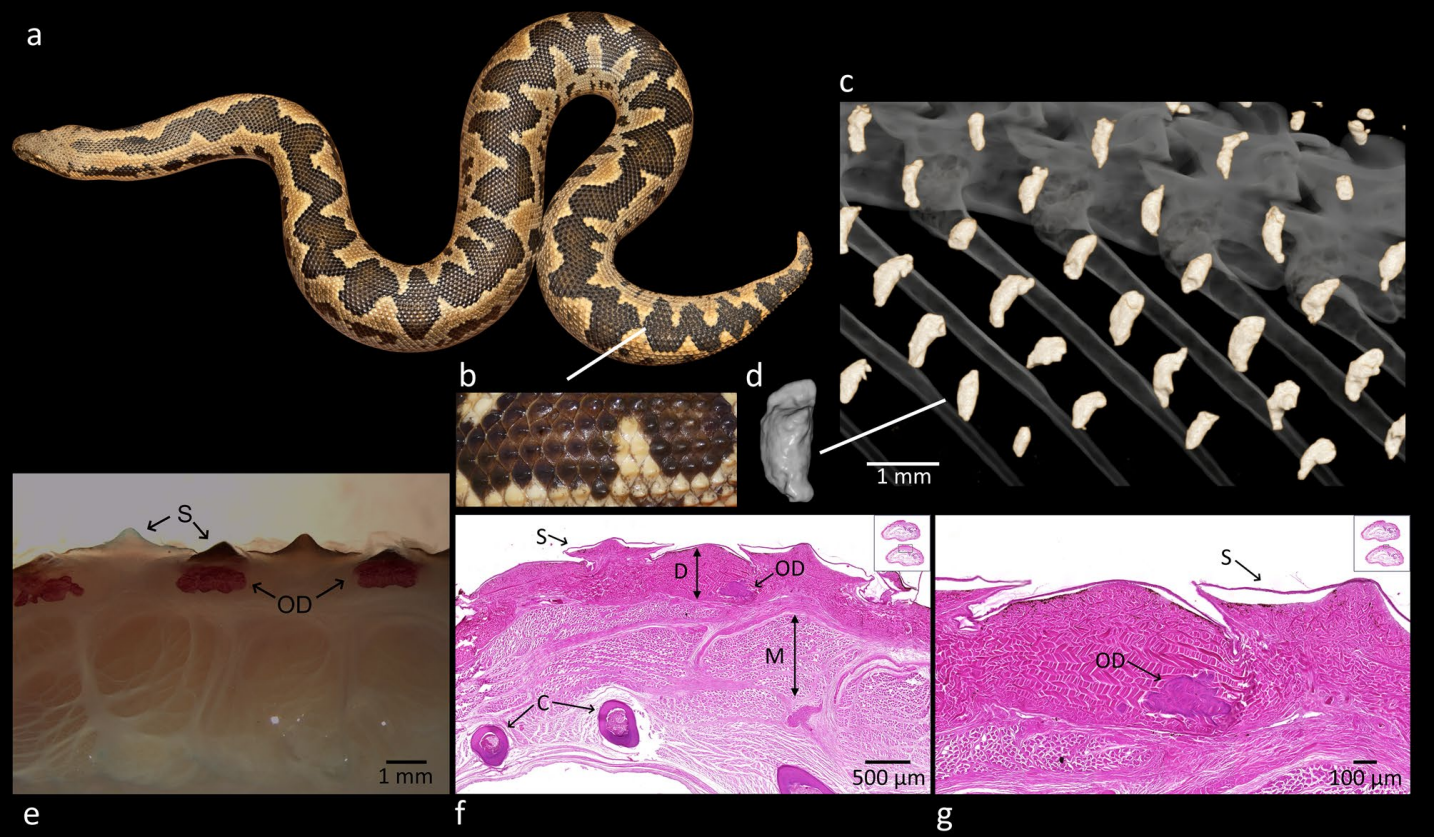
Structure and composition of the osteoderms of Rough-tailed sand boa (*Eryx conicus*). (**a**) Whole-body photo of a “two-headed” snake; (**b**) the detail of rough scales on the top of the tail; (**c**) reconstructed µCT scan of lateral dermal armour and axial skeleton with ribs; (**d**) the detail of osteoderm; (**e**) osteoderms from the lateral dermis of the caudal part of the body prepared as a whole-mount using Alizarin red (single-stained); (**f**) transverse section (dorsal towards the top) of the caudal part of the body stained by haematoxylin and eosin; (**g**) the detail of osteoderm from transverse section of the caudal part of the body stained by haematoxylin and eosin. Note in (**c**) vermiform-like osteoderms (yellow), while the axial skeleton and ribs are grey. Osteoderms in (**e**) resides entirely within the pars papillaris corii closely below the squama. The origin of our samples (private collections of animals died of natural causes) prevents us to preserve the tissue for fine histology. The nuclei in (**f**, **g**) are not visible because they were already degraded. OD (osteoderm), C (costae), D (dermis), M (musculus), S (squama). Author: Petra Frýdlová, Jan Dudák, Milada Halašková, Markéta Riegerová and Daniel Frynta.

Given that dermal armour appears across all lepidosaur lineages, its purported total absence in snakes with over 3500 species^53^ is somewhat questionable and can be explained by the necessity of maintaining the body flexibility of legless snakes. Dermal armour reduces agility and movement speed as was demonstrated in cordylids^54^. On the other hand, the legless Slow worm lizard (*Anguis fragilis*) or European legless lizard (*Pseudopus apodus*), which have armoured the whole body^54,55^, does not seem to have a problem with rapid movement. Thus, we suspected that there might be some snake species with well-developed dermal armour. To narrow down the number of species to examine, we focused on groups with a specific antipredatory strategy and ecology. In the end, we selected sand boas from the family Erycidae (superfamily Booidea), a group of fossorial snakes that perform tail-displays^56^, during which the tail becomes unusually conspicuous in response to stress or tactile stimuli. Sand boas live secretively, hidden in the sand most of the time^57^. They use tail displays as a successful antipredatory strategy^56^ to avoid serious injuries to vulnerable body parts (e.g., the head). Their tail is usually difficult to distinguish from the head as it has the same shape, giving rise to the moniker “two-headed snakes”^58^ (Fig. 1a). The tail is rough and hard to the touch in some species (Fig. 1b). This pattern is common in species with dermal armour (e.g., scincoid and anguid lizards), which also supports our interest to search for dermal armour in this group. The morphology of their caudal vertebrae is highly modified^59,60^, featuring excessively bifurcated neural spines, unique accessory lateral processes, well-developed lateral processes, and other peculiarities. To our knowledge, this unique morphology has not been associated with antipredatory (or other) functions in the literature.

In this study, we aimed to investigate tail morphology in fossorial snakes and snakes with tail displays to search for dermal armour. We employed µCT imaging to detect potential dermal ossifications. In addition to sand boas, we have supplemented our dataset with comparative material from boas, pythons and other snake groups.

## Results

**Figure 2:**
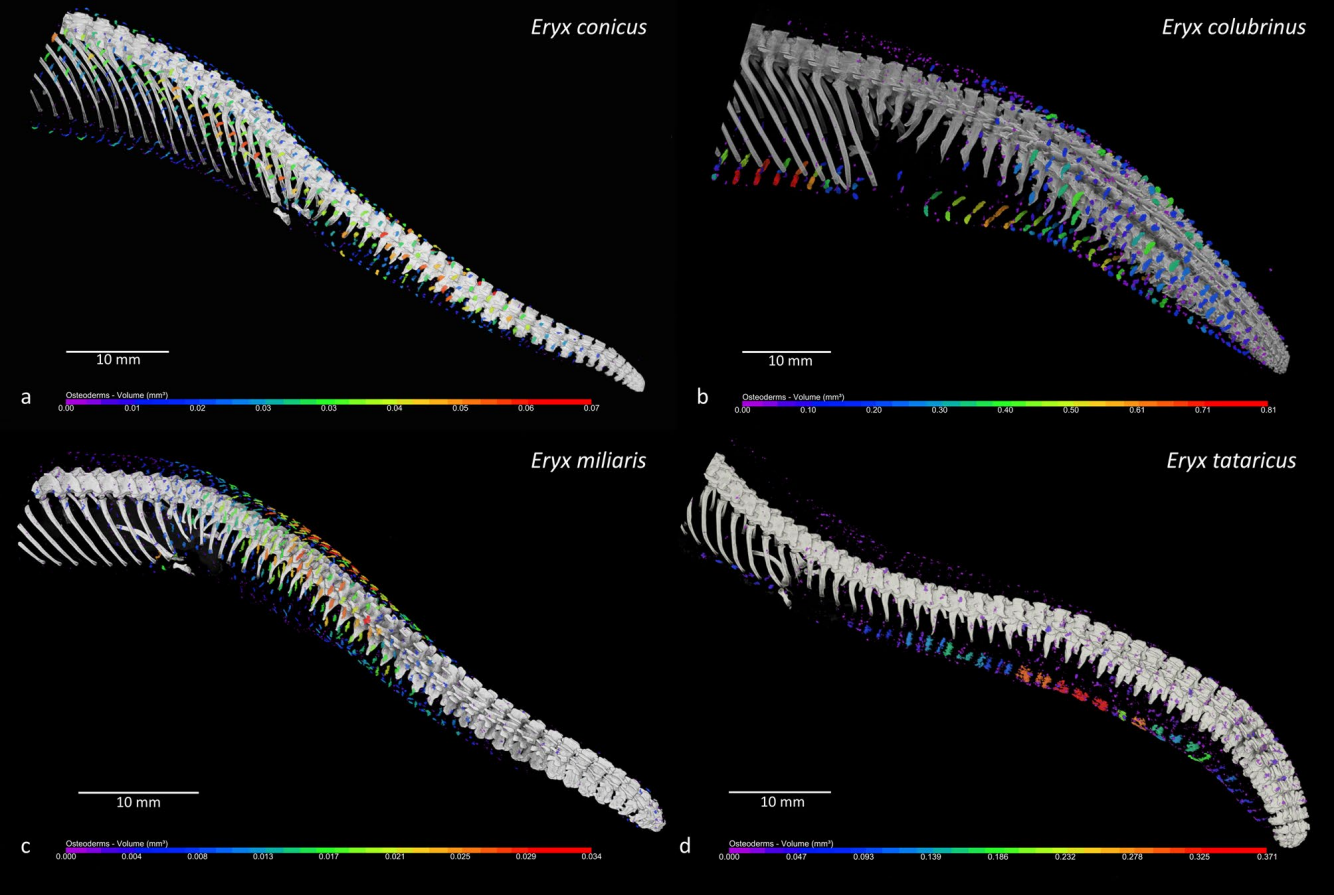
Visualisation of the caudal part of the body and tail of the sand boas by µCT. (**a**) *Eryx conicus*, (**b**) *E. colubrinus*, (**c**) *E. miliaris*, (**d**) *E. tataricus*. The small, coloured structures are osteoderms, which are present on the tail and the caudal part of the body anterior to the cloaca. Colours are according to the volume (in mm^3^) of osteoderms. Note the different scales of volume for each species. Osteoderms do not cover the body continuously; rather they are individually distributed across the surface inside the skin. The distribution of osteoderms is regular resembling the distribution of scales. Bar 10 mm. Author: Petra Frýdlová and Jan Dudák.

In total, we examined 68 specimens belonging to 27 snake species (Table 1). We found dermal armour in four species of sand boas by µCT. It was present on the tail and the body around four cm anterior to the cloaca and its distribution was rather regular resembling the distribution of scales. On the dorsal and lateral sides, it copies the pattern of scale rows (Fig. 1c). On the ventral part it has the shape and location corresponding with ventral scales (Fig. 2b). The position of osteoderms is closely below scales (Fig. 1e) suggesting that each scale in the lower part of the body is supported with osteoderm. The most typical shape of dorsal and lateral osteoderms was vermiform-like (Fig. 1c, d) as described in Komodo dragons^39^, but their density was not so high. Ventral osteoderms were often rod-like shaped (Fig. 2b). Nevertheless, the variability in the shape of osteoderms was high, especially in the small specimens. It is most probably due to its ongoing development. Histological analysis (haematoxylin and eosin staining) revealed small-mineralised osteoderms (367 × 167 µm in diameter) incorporated into the dermis (Fig. 1f, g). Alizarin staining sensitive to mineralised tissues revealed the same result. Transversal sections uncovered small osteoderms close to the surface of the skin incorporated in the superficial dermis right below scales (Fig. 1e).

**Table 1:** The list of studied specimens in alphabetical order with details about the sex, age, presence of osteoderms, caudal vertebrae modifications, ecology, body size and relative body size.

Species	ID	Sex	Age (years)	ODs	CVM	Fossorial	SVL (cm)	TL (cm)	TOL (cm)	SVLrel (%)
*Acrantophis madagascariensis*	683	♀	A (> 15 y)	0	0	NF	220	15.1	235.1	97.96
*Boa imperator*	686	♂	A (> 10 y)	0	0	NF	106	17	123	91.07
*Calabaria reinhardtii*	667	♀	A (> 10 y)	0	0	F	60	6	66	77.32
*Candoia aspera*	359	♀	A	0	0	NF	60	1.5	61.5	102.04
*Candoia carinata*	425	♂	A (> 20 y)	0	0	NF	63.5	8	71.5	137.98
*Chilabothrus angulifer*	355	♂	A	0	0	NF	125	18.6	143.6	78.06
*Corallus hortulanus*	426	♂	A (> 20 y)	0	0	NF	123.3	27.1	150.4	100.00
*Epicrates maurus*	400	♀	A	0	0	NF	89.5	13.2	102.7	75.21
*Eryx colubrinus*	662	♂	A	0	1	F	30.2	3.2	33.4	77.46
*Eryx colubrinus*	663	♀	A	0	1	F	48	3.5	51.5	91.06
*Eryx colubrinus*	670	♂	A	1	1	F	37.6	4.7	42.3	96.43
*Eryx colubrinus*	674	♂	JUV	0	1	F	19.5	2	21.5	50.01
*Eryx colubrinus*	689	♂	A	0	1	F	53.5	2.9	56.4	137.21
*Eryx colubrinus*	695	♀	A	1	1	F	58.1	5	63.1	110.23
*Eryx colubrinus*	696	♀	A	1	1	F	49	4.7	53.7	92.96
*Eryx colubrinus*	703	♀	A	0	1	F	43.3	4.7	48	82.15
*Eryx colubrinus*	706	♂	A	0	1	F	32.4	3	35.4	83.10
*Eryx colubrinus*	710	♂	A	0	1	F	34	4.2	38.2	87.20
*Eryx colubrinus*	712	♂	SA	0	1	F	26	2.5	28.5	66.68
*Eryx colubrinus*	714	♀	SA	0	1	F	27.6	2.1	29.7	52.36
*Eryx colubrinus*	719	♀	SA	0	1	F	27.5	2.1	29.6	52.17
*Eryx colubrinus*	720	♂	SA	0	1	F	24.7	2.8	27.5	63.35
*Eryx colubrinus*	731	♀	A	0	1	F	41.8	3.4	45.2	79.30
*Eryx conicus*	428	♀	A	1	1	F	72.5	5.5	78	137.21
*Eryx conicus*	661	♂	A	1	1	F	36.5	4	40.5	112.62
*Eryx conicus*	671	♀	A	1	1	F	48.7	4.4	53.1	92.17
*Eryx conicus*	672	♀	SA	1	1	F	31.6	2.8	34.4	59.80
*Eryx conicus*	680	♀	A	1	1	F	49.5	3.9	53.4	93.68
*Eryx conicus*	681	♀	A	0	1	F	32	2	34	60.56
*Eryx conicus*	704	♀	JUV	0	1	F	14.5	1.1	15.6	27.44
*Eryx conicus*	707	♀	JUV	0	1	F	14.9	0.8	15.7	28.20
*Eryx conicus*	729	NA	SA	0	1	F	39.3	2.6	41.9	–
*Eryx conicus*	730	♀	A	1	1	F	66.9	5.1	72	126.61
*Eryx conicus*	733	♂	A	1	1	F	39	3.3	42.3	120.33
*Eryx jaculus*	735	♂	A	0	0	F	32.4	4.6	37	91.27
*Eryx johnii*	469	NA	JUV	0	1	F	24.6	1.1	25.7	–
*Eryx johnii*	471	NA	JUV	0	1	F	22	2.1	24.1	–
*Eryx johnii*	664	♂	A (> 20 y)	0	1	F	53.5	6.5	60	89.48
*Eryx johnii*	721	♂	A (> 20 y)	0	1	F	63.5	9.2	72.7	106.21
*Eryx miliaris*	678	♂	A (> 10 y)	0	1	F	46	6	52	149.84
*Eryx miliaris*	679	♀	A (> 10 y)	1	1	F	36.5	5.2	41.7	119.28
*Eryx muelleri*	665	♀	A (> 8 y)	0	1	F	56	4.6	60.6	140.14
*Eryx muelleri*	676	♂	A (> 8 y)	0	1	F	53.7	4.2	57.9	177.40
*Eryx muelleri*	677	♂	A (> 8 y)	0	1	F	51.6	3.3	54.9	170.47
*Eryx muelleri*	678	♂	A (> 8 y)	0	1	F	48	4.5	52.5	158.57
*Eryx muelleri*	688	♀	A (> 8 y)	0	1	F	54.6	4.5	59.1	136.64
*Eryx muelleri*	700	♀	A (> 8 y)	0	1	F	55.7	4.3	60	139.39
*Eryx tataricus*	666	♂	SA	0	1	F	32	3	35	100.00
*Eryx tataricus*	673	♀	A (> 15 y)	1	1	F	42.8	5.8	48.6	133.75
*Eryx tataricus*	675	♂	A	0	1	F	28.6	5.1	33.7	89.38
*Eryx tataricus*	701	♀	A	0	1	F	39	2.4	41.4	121.88
*Eryx tataricus*	702	♂	A	0	1	F	46.3	3.4	49.7	144.69
*Eryx tataricus*	705	♀	SA	0	1	F	16.3	2.7	19	50.94
*Eryx tataricus*	709	♂	A	0	1	F	30.4	4.3	34.7	95.00
*Eunectes murinus*	687	♂	A (> 10 y)	0	0	NF	147	25	172	77.00
*Gonionotophis poensis*	665	♂	A	0	0	NF	106	16.7	122.7	98.15
*Hemorrhois ravergieri*	431	♂	A	0	0	NF	86.3	26	112.3	108.55
*Lampropeltis ruthveni*	528	♂	A (> 10 y)	0	0	NF	141.7	17.7	159.4	125.51
*Liasis mackloti*	684	♀	A (> 15 y)	0	0	NF	156	29	185	76.17
*Lichanura trivirgata*	668	♀	SA	0	0	SF	32.5	8	40.5	52.93
*Lichanura trivirgata*	722	♂	A (> 10 y)	0	0	SF	57.6	8.7	66.3	103.78
*Python regius*	561	♂	A	0	0	NF	100.7	10.9	111.6	90.48
*Thamnophis sirtalis*	493	♀	A	0	0	NF	63.1	20.6	83.7	122.52
*Tropidophis melanurus*	723	♀	A (> 20 y)	0	0	NF	79	8.4	87.4	80.11
*Tropidophis melanurus*	724	♂	A (> 20 y)	0	0	NF	62.8	6	68.8	75.66
*Tropidophis pardalis*	496	♂	A	0	0	NF	19.5	3.1	22.6	88.64
*Xenopeltis unicolor*	670	♂	A	0	0	SF	69	7.1	76.1	132.69
*Xerotyphlops vermicularis*	737	♀	A	0	0	F	20.5	0.5	21	100.05

ID, identity of the specimen; ODs, osteoderms; CVM, caudal vertebrae modifications; SVL, snout-vent length; TL, tail length; TOL, total body length; SVLrel, relative body size; ♀, female; ♂, male; 0, absent; 1, present; F, fossorial; SF, semi-fossorial; NF, not fossorial; A, adult; SA, subadult; JUV, juvenile.

We found clearly discernible dermal armour in all but one of the seven adults of the Rough-tailed sand boa (*Eryx conicus*) and one subadult animal. Dermal armour was present on the tail and the body around four cm anterior to the cloaca (Table 1, Fig. 2a; Supplementary video 1; SI1, SI6). Osteoderms were the densest and bulkiest on the dorsal and lateral part of the tail, which is in line with the putative antipredatory function of dermal armour. Osteoderms were also present on the ventral part. They did not cover the tail continuously; rather they were individually distributed across the surface in a regular fashion, resembling the distribution of scales. The mean volume of osteoderms was 1.60E−02 mm^3^ (range 9.74E−07–0.07 mm^3^).

Dermal armour was present also in the Kenyan sand boa (*E. colubrinus*). However, it was well developed only in one out of ten adult specimens (Fig. 2b; Supplementary video 2; SI1, SI5). Mean volume of osteoderms was 0.07 mm^3^ (range 9.93E−04–0.81 mm^3^). In two other adult individuals, it was much less developed, with only small osteoderms present on the ventral part of the tail. We also found dermal armour in one adult specimen of *E. miliaris*, which was mostly placed on the basal part of the tail (Fig. 2c; Supplementary video 3; SI1, SI7). In one adult *E. tataricus*, osteoderms were present on the whole ventral part of the tail (Fig. 2d; Supplementary video 4; SI1, SI8). The mean volume of osteoderms in *E. miliaris* and *E. tataricus* was 4.19E−03 mm^3^ (range: 1.46E−04–3.35E−02 mm^3^) and 5.05E-03 mm^3^ (range 1.45E−04–0.37 mm^3^), respectively. We did not find any osteoderms in juveniles and subadults (except *E. conicus*). The volume of dermal armour is positively correlated with the body size and age of the specimen. This suggests that osteoderms develop across ontogeny in adulthood in sand boas.

**Figure 3:**
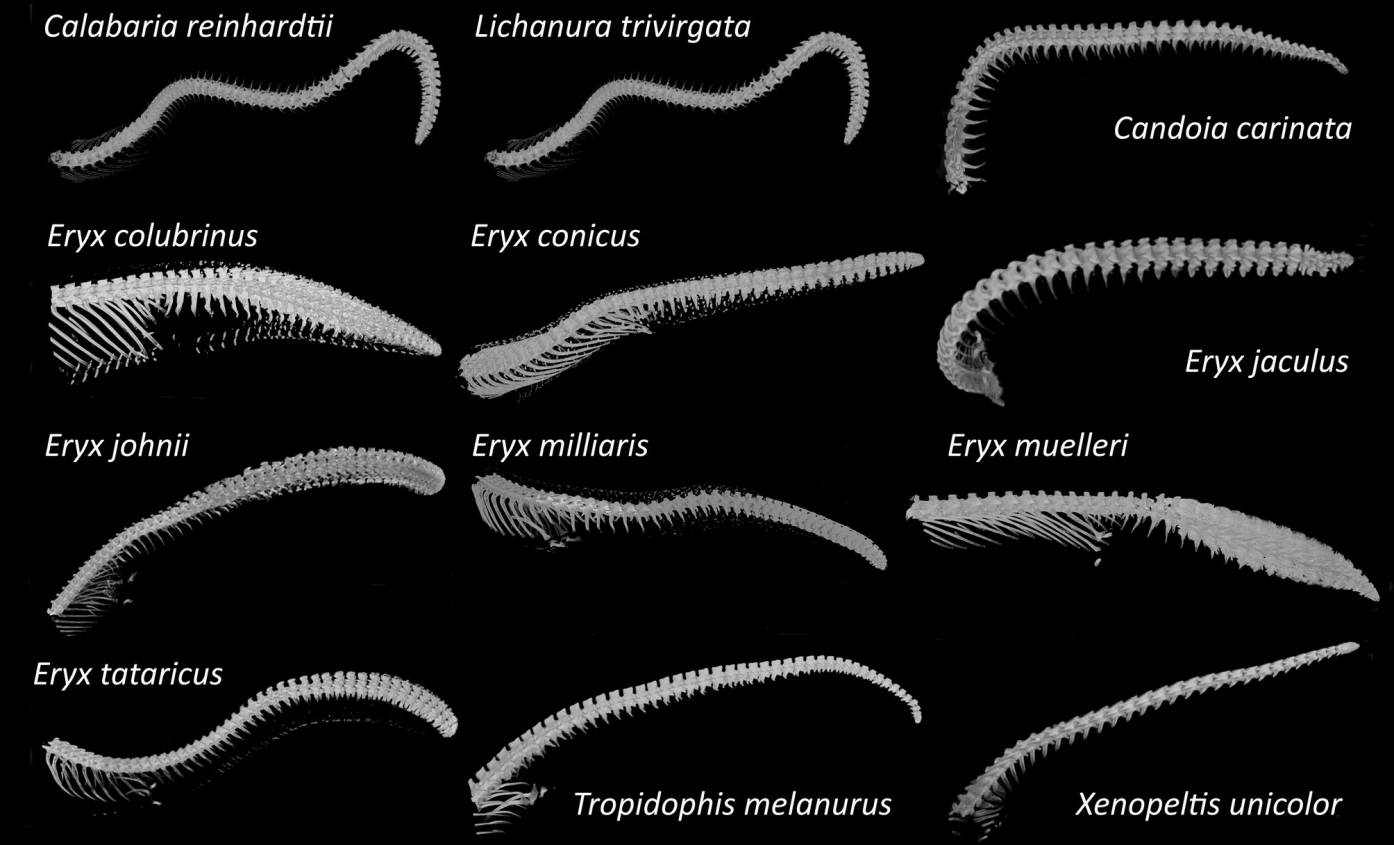
Caudal and tail morphology of studied snakes as revealed by µCT. Note small osteoderms visible in *E. colubrinus*, *E. conicus*, *E. miliaris* and *E. tataricus.* Caudal vertebrae modifications are apparent in all species of *Eryx* except *E. jaculus*. Author: Petra Frýdlová and Jana Mrzílková.

**Figure 4:**
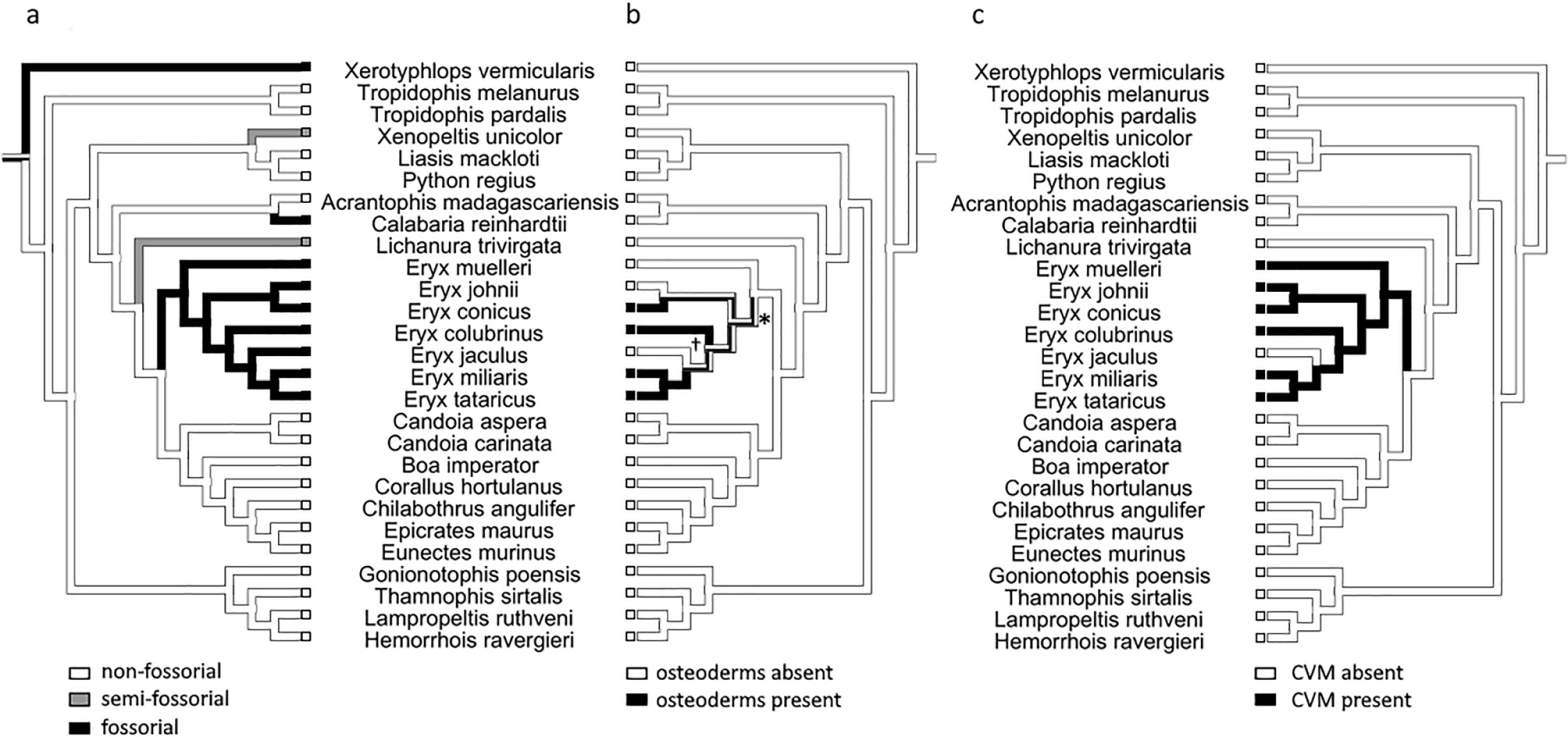
Visualisation of ecology, osteoderms and caudal vertebrae modifications on the phylogenetic tree. (**a**) fossoriality, (**b**) presence of osteoderms, and (**c**) presence of caudal vertebrae modifications (CVM) in snakes. Ancestral state reconstruction using maximum parsimony following the topology of Reynolds et al. 2014 was employed in Mesquite. Abbreviations: * the putative origin of dermal armour in sand boas, † the possible uncertainty of presence/absence of osteoderms due to the low sample size of *E. jaculus.* Author: Petra Frýdlová.

We failed to detect similar dermal armour in the other examined sand boas. In *E. jaculus*, it can be due to the low sample size. In *E. johnii* was sample size also small, nevertheless, those individuals were fully-grown and more than 20 years old. *E. muelleri* were fully-grown and the sample size was sufficient. Nevertheless, we confirmed the presence of highly pronounced modifications of the caudal vertebrae in sand boas (Fig. 3), not found in any other studied species. In the rest of the studied species, we found neither osteoderms nor modified caudal vertebrae. Tail vertebrae were rather uniform and decreased in volume in the caudal direction, even in species in which tail displays have been described (Fig. 3). Based on these results, we hypothesise that osteoderms and modifications in caudal vertebrae are unique to sand boas and that they might be associated with fossorial ecology and a specialized foraging tactic (prey on rodent litters in burrows). To evaluate this hypothesis, we performed ancestral state reconstruction on a mirror tree (tree topology adopted from Reynolds et al.^61^) using maximum parsimony as implemented in Mesquite (Fig. 4). The most parsimonious scenario is one origin of dermal armour in the last common ancestor of all *Eryx* species except *E. muelleri* (asterisk in Fig. 4b) and later two independent losses of this armour in *E. johnii* and *E. jaculus*. An alternative scenario suggests three independent origins of dermal armour. Other scenarios are less parsimonious, e.g., if the dermal armour is an apomorphy of the entire genus *Eryx,* one more loss in *E. muelleri* is required. The results were virtually the same for an alternative tree topology and ML method (see SI 2–4).

Caudal modifications are present in all members of the genus *Eryx* (except *E. jaculus*), thus a single origin in the ancestor of this group is the most parsimonious scenario (Fig. 4c). We consider both characters to be likely morphological innovations exclusive to Erycidae and adaptive for fossorial snakes in an antipredatory context.

## Discussion

We described for the first time the presence of dermal armour in snakes. Osteoderms were discovered on the caudal part of the body (mostly on the tail) in four species of sand boas. They were present mostly in adult specimens of both sexes in well-represented species (*E. conicus* and *E. colubrinus*). In *E. tataricus*, we found them only in one very old female. The volume of dermal armour seems to be positively correlated with the body size and age of the studied specimen. The position of osteoderms is in the surface layer of the dermis closely below scales and it copies the pattern of dorsal, lateral, and ventral scales on the lower part of the body. It is most probably responsible for the passive tail protection as well as for the reduction of tail skin flexibility. The shape of osteoderms is highly variable, which is typical for this type of skeletal element in squamates^29^. The most common shape is vermiform-like resembling those described in Komodo dragons (*Varanus komodoensis*)^39^.

Osteoderms are common in squamates^31,62^, nevertheless, the specific phylogenetic distribution is highly irregular^1^. Our data on sand boas indicate a similarly irregular pattern, as they are not present in all the studied species (Fig. 4b). Absence in *E. jaculus* can be associated with low sample size. Nevertheless, even if it will be later uncovered, it will not change the scenario of one independent origin. Repeated independent origins and/or losses of osteoderms support the idea of the latent plesiomorphic ability of the dermis to form this structure if needed^11,25^.

We propose an antipredatory function of osteoderms in sand boas based on (1) the caudal distribution on the body, (2) fossorial ecology, and (3) the caudal vertebrae modifications. Sand boas are fossorial snakes with rather short, stout bodies. The tail resembles the head, which, in combination with the tail displays, causes the attention and attacks of predators to be directed to this less vulnerable part of the body, the tail. This was supported by Greene^56^, who studied the prevalence of tail injuries in different species of snakes employing tail displays (especially *E. johnii* and *Charina bottae*). We hypothesise that the additional protection of this part of the body with dermal armour can be highly adaptive. Later, Hoyer^63^ suggested that injuries in *C. bottae* primarily result from prey protecting their pups (specifically, parents of altricial rodents). Accordingly, the dermal armour reinforcing the tail tip evolved to withstand repeated attacks not from predators but from prey. We support this latter hypothesis. Sand boas forage on rodent litters in burrows (for a thorough review of sand boas' prey, see^64^). They enter the burrow through narrow underground passages and prey in the nest. We hypothesise that when the parents return to the nest and discover the snake intruder, they direct their attacks to the tail, which is the first object they meet on their way to the nest. At the same time, the snake is not able to defend itself by biting due to insufficient space for turning around inside the passage. Thus, we think that they need to protect their tail passively, with dermal armour. We compare this armour to the brigandine armour of medieval warriors. Our hypothesis is also supported by the unique caudal vertebrae modifications in this clade. In most sand boa species, the tail is filled with enlarged vertebrae (Fig. 3, and references^59,60^). The ancestral state reconstruction revealed one origin of caudal vertebrae modifications in Erycidae. Even though we also examined several other fossorial and semi-fossorial (categorization according to^65^) snake species (e.g., *Xerotyphlops, Calabaria, Xenopeltis*), we did not find similar morphological adaptations (neither osteoderms nor caudal vertebrae modifications), not even in the closely related *Lichanura*. The common absence of dermal armour in snakes can be explained by the trade-off between flexibility and armour. Exaggerated dermal armour is usually associated with the reduction of speed and manoeuvrability^54^. While sand boas are rather slow snakes^66^ and the distribution of dermal armour is on the posterior part of the body, the protective advantage (presence of armour) can offset the disadvantages (flexibility and speed reduction). To explore the functional performance of the dermal armour and its adaptivity in sand boas, it will be necessary to test it (e.g., by the comparison of skin penetrability on armoured and non-armoured parts of the body). Nevertheless, current function of a certain structure does not provide sufficient evidence about the adaptive history of this character. It may originally evolve due to a different kind of selective pressure (preadaptation).

The uncovered evolution of osteoderms and unique caudal vertebrae modifications in sand boas supports the general tendency of fossorial species to adopt highly specialized morphology, physiology, diet, and locomotion (summarized in^67^). Several other morphological adaptations have been revealed in sand boas, such as specialized scales^68^ resistant to abrasion in sandy habitats^69^, a wedge-shaped digging snout^70^, and thin and small spectacles^71^. Fossorial reptiles often exhibit miniaturization of the skull and highly specialized cranial osteology associated with burrowing^72,73^, which also imposes dietary restrictions and specializations^64,74,75^. A recent analysis focused on squamates tested whether fossoriality, a trait that promotes specialization in many different aspects, acts as an evolutionary dead-end^65^. Phylogenetic comparative methods revealed that transition rates from fossoriality to non-fossoriality are significantly lower than vice versa. Moreover, they also showed that fossoriality is an evolutionary dead-end for snakes but not for lizards.

Sand boas have a relatively rich fossil record from localities in Eurasia and the Middle East, which dates back to the Eocene^60,76^. Their place of origin has been disputed; nevertheless phylogenetic scenarios suggested that the clade radiated initially in the Old World^77^. A nearly complete skeleton of the oldest erycine-like boid (*Rageryx schmidi*) from Germany is dated to early-middle Eocene^70^. A thorough morphological examination revealed distinctive accessory processes on caudal vertebrae, which were not as elaborate as in extant species. Moreover, the premaxilla was not so wedge-shaped, and the inner ear lacked the typical adaptations to a burrowing life mode. They do not report the presence of dermal armour, but this structure can be hardly preserved in the fossil record. Smith and Scanferla^70^ concluded that this ancestor of Erycidae and Charinidae was not fossorial. Based on the available fossil record from the Miocene^71^ and middle Pliocene, Szyndlar and Schleich^60^ suggest that most representatives of European Erycidae were similar to the extant Asian members of the genus with well-developed caudal accessory processes^60^ and were most probably fossorial.

The phylogeny of the superfamily Booidea is still disputed, especially the position of *Calabaria reinhardtii*^78,79^, the position of *Candoia*, the question of monophyly of the family Erycidae with the genera *Eryx*, *Lichanura, Ungaliophis, Exiliboa*, and *Charina*, and the relationships inside sand boas. We decided to follow the more conservative phylogeny of boas and pythons from Reynolds et al.^61^. Nevertheless, the results were comparable when the fully sampled phylogeny from Tonini et al.^80^ was used, as the studied characters (osteoderms, caudal morphology) are present only inside the clade Erycidae. These two phylogenies also differ in the position of *E. colubrinus* and *E. jaculus* inside sand boas. We prefer the position of *E. jaculus* from Reynolds et al.^61^ as it is congruent with the new molecular data for the Iranian populations of sand boas^81^. The position of *E. colubrinus* is still problematic, as evident from the support values in Reynolds et al.^61^. Despite these uncertain relationships inside sand boas, we uncovered the same evolutionary scenarios concerning the origin of dermal armour using both phylogenies^61,80^ (SI 10–12).

The distribution of dermal armour within sand boas is irregular. The absence in *E. jaculus* can stem from the rather low sample size. Nevertheless, adult specimens of *E. johnii* were fully-grown and old individuals (> 20 years old). As the volume of osteoderms is positively correlated with age and body size, it should be already present in those individuals. In *E. muelleri* we examined six adult fully grown specimens. Despite this higher sample size, we did not detect any evidence of dermal armour in this species. From an evolutionary point of view, it is interesting that *E. muelleri* is the most basal sand boa (its position is stable across various phylogenies). Moreover, its tail exhibits the most elaborated vertebral processes (Fig. 3). We can speculate about the coevolution and a possible trade-off between caudal modifications and dermal armour, which both play an important role in antipredator defence. The species with the most developed dermal armour (*E. conicus*) considerably reduced the volume of caudal vertebral processes, supporting this putative trade-off.

We were quite surprised that we did not find dermal armour in the Calabar burrowing python (*C. reinhardtii*), as its body is very hard to the touch (like in gerrhosaurids, cordylids, and anguids). Nevertheless, a detailed analysis of the mechanical properties of their skin^82^ revealed a highly structured lamellar arrangement of the dermal collagen bundles, markedly thick integumentary layers, and a reduction of the hinge region in between the scales, which in mutual combination provide enormous penetration resistance. The authors hypothesised that these skin properties of Calabar pythons serve as a passive defensive mechanism against penetrative bites from maternal rodents and predators. If true, it would be an example of the convergent evolution of a passive defensive strategy based on two different morphological structures in the skin in species with similar ecology (fossoriality, specialization on rodent pups). On the other hand, the absence of dermal armour in *Xerotyphlops vermicularis* was not so surprising. These small-bodied secretive snakes are fossorial. Due to their specialised diet (myrmecophagy), they stay close to anthills and termite mounds, which offer a passive defence. Thus, the evolution of additional protective morphological structures would be redundant.

We can consider other candidate snake species, which might have developed dermal armour. It would be especially useful to sample the remaining closely related species belonging to Erycidae and Charinidae. Moreover, it would be interesting to examine other fossorial species employing tail displays and specialising in picking rodent pups (e.g., *Uropeltis, Cylindrophis, Loxocemus, Calamaria*). We checked available material on MorphoSource.org^83^ but did not find osteoderms in any of the above-mentioned genera.

In conclusion, we discovered covert dermal armour in sand boas. We hypothesise this passive defensive strategy is associated with fossoriality and a specialized foraging tactic. Our hypothesis is supported by the specialized skeletal morphology of the tail, with highly modified caudal vertebrae nearly filling in the whole volume of the tail.

## Methods

### Specimens

We examined the tails of formalin- or ethanol-preserved specimens from the private collections of D.F. and P.F. We focused on the genus *Eryx* (seven species), recently revised by Reynolds and Henderson^84^, and included other genera of the superfamily Booidea (10 species). We also supplemented the sample with 10 additional species from different snake families (see Table 1 for details) as outgroups. We examined 68 specimens in total, 56 specimens with µCT and 12 specimens with micro-radiography. Those techniques are suitable for visualisation of hard tissues. In comparison to the histology, they are not so sensitive to the preservation of samples and allow to inspect larger sample size.

We measured total body length (TOL), snout to vent length (SVL) and tail length (TL) of all specimens (Table 1). We completed our data with relative body size (SVL_rel_) in % computed as SVL of specimen/mean SVL typical for the species and sex*100 (data for mean SVLs came from the literature, for data and references, see Tab. S1 in SI1). Since 75% is the mean relative size at maturity found in squamates^85^, we consider animals with SVL_rel_ > 75% as adults. We were able to collect many fully or nearly fully-grown animals with SVLrel > 90%. We cut off the tail in the area above the cloaca to fit the sample into a 60 ml test tube. In seven selected species (*E. conicus*, *E. colubrinus*, *E. johnii*, *E. muelleri*, *E. tataricus*, *Calabaria reinhardtii*, and *Lichanura trivirgata*), we examined the entire body to search for dermal armour.

### Imaging techniques

The µCT analysis was carried out using two different cone-beam micro-CT scanners—Bruker SkyScan 1275 (Bruker microCT, Kontich, Belgium), situated at the Specialized Laboratory of Experimental Imaging, and a custom-built µCT scanner operated at the micro-CT laboratory of the Institute of Experimental and Applied Physics^86^.

The SkyScan 1275 is well suited for routine and rapid scanning of larger sample sets, but the maximum achievable spatial resolution is insufficient in some cases. It was, therefore, used for initial preview scans of the complete sample set. Each measurement consisted of several cone-beam scans that were merged into a single volume during the tomographic reconstruction. This technique is an in-built feature of the scanner and allows for scanning objects exceeding the detector field of view in the axial direction. The tube was operated at 40 kVp and 250 µA. Each scan consisted of 1800 equidistant projections. The projection data were reconstructed using the Bruker Nrecon software. The effective pixel size was set within the range of 26 to 51 µm, depending on the size of individual samples.

Based on the obtained results, a subgroup of specimens (with detected osteoderms) was selected for a more detailed scan using the custom-built scanner. This machine allowed us to prepare high-resolution pictures and animations for publication. The system was equipped with an X-ray tube Hamamatsu L12161-07 and an X-ray imaging detector Dexela 1512. The tube is a sealed-type micro-focus source with a tungsten target and provides an acceleration voltage up to 150 kV and a tube current of up to 500 µA. The detector is an active-pixel CMOS flat panel equipped with a 200 µm thick micro-columnar CsI scintillation sensor and a 1944 by 1536 pixelated read-out array with a pitch of 74.8 µm. The helical scanning geometry was utilized given the elongated shape of the measured samples. The tube was operated at 60 kVp and 150 µA. The spectrum was filtered with a 1 mm thick aluminium filter. The beam pitch was set to 0.5 and the projection angle step to 0.6°. The achieved effective pixel size varied within the range of 8–15 µm depending on the dimensions of each sample. The CT reconstructions were carried out in Volume Graphics Studio MAX (Volume Graphics GmBH, Heidelberg, Germany) using an iterative algebraic reconstruction technique included in the dedicated CT-reconstruction module. The data analysis, segmentation and visualization were performed using Bruker CTVox (Bruker microCT, Kontich, Belgium) and Dragonfly software^87^. The segmentation procedure consisted of two steps. First, the bone-like structures were selected by a threshold using the Otsu algorithm. Then, the Connected component Analysis—a built-in tool of the Dragonfly software—was used to separate the individual non-touching objects obtained from the thresholding procedure. After that, all segmented osteoderms can be handled as individual objects and their properties like dimensions, volume, shape, and position in space can be analysed.

We completed our study with histological methods. We examined samples from *E. conicus* to search for mineralised tissue. We removed a piece of the skin transversally from the caudal part of the body (3 cm anterior to the cloaca). Samples were fixed in 100% ethanol, transferred to 1.5% potassium hydroxide for digestion, and stained by the solution of 0.001% Alizarin in 1% potassium hydroxide. Then the samples were washed in distilled water and processed in ascending series of glycerol solutions in water and stored in 100% glycerol. Samples were examined under a stereo magnifier.

To examine the tissues in more detail, we also prepared histological sections. We separated 2 cm of the caudal part of the body (2 cm anterior to the cloaca) from *E. conicus*. The samples were fixed in 96% ethanol and decalcified in a mixture of equal parts of formic acid 40% and sodium-citrate 20%^88^. The sample was dehydrated in graded ethanol series and embedded in paraffin. The whole body was transversally cut into 7 μm sections by a microtome. The sections were stained with haematoxylin and eosin. The slides were examined under a binocular microscope.

### Ancestral state reconstruction

Ancestral state reconstruction was performed in Mesquite v. 3.7.0^89^ using maximum parsimony and maximum likelihood (ML). For the phylogenetic relationships, we used a species-level molecular phylogeny for boids and pythons from Reynolds et al.^61^ and a comprehensive squamate phylogeny from Tonini et al.^80^. We scored osteoderms as present, when they were visible as isolated high-density bone-like structures in the dermis, completely separated from the skeleton. The caudal modifications were scored as present when there were excessively bifurcated neural spines, unique accessory lateral processes, and/or well-developed lateral processes.

## Supplementary Information

Supplementary Information 1.

Supplementary Information 2.

Supplementary Information 3.

Supplementary Information 4.

Supplementary Information 5.

Supplementary Information 6.

Supplementary Video 1.

Supplementary Video 2.

Supplementary Information 7.

Supplementary Video 3.

Supplementary Information 8.

Supplementary Video 4.

## Data Availability

All data generated or analysed during this study are included in this published article (and its Supplementary Information files) and on Dryad repository (https://doi.org/10.5061/dryad.fxpnvx0wg).
