# Supplementary material for: The first description of dermal armour in snakes
Source: Sci Rep. 2023 Apr 19;13:6405. doi: 10.1038/s41598-023-33244-6 (PMC10115820; doi:10.1038/s41598-023-33244-6)
Supplement: Supplementary file 1 — Supplementary Information 1. [file 41598_2023_33244_MOESM1_ESM.docx]

**Supplementary Informations 1**

**The first description of dermal armour in snakes**

Petra Frýdlová, Veronika Janovská, Jana Mrzílková, Milada Halašková, Markéta Riegerová, Jan Dudák, Veronika Tymlová, Jan Žemlička, Petr Zach, Daniel Frynta

**Captions for supplementary figures and videos**

**SI 2**. Figure. Visualisation of (a) ecology, (b) presence of osteoderms, and (c) presence of caudal vertebrae modifications (CVM) in snakes. Ancestral state reconstruction using maximum parsimony following the topology of Tonini et al. 2016 was employed in Mesquite. Author: Petra Frýdlová.

**SI 3**. Figure. Visualisation of (a) ecology, (b) presence of osteoderms, and (c) presence of caudal vertebrae modifications (CVM) in snakes. Ancestral state reconstruction using maximum likelihood following the topology of Reynolds et al. 2014 was employed in Mesquite. Author: Petra Frýdlová.

**SI 4**. Figure. Visualisation of (a) ecology, (b) presence of osteoderms, and (c) presence of caudal vertebrae modifications (CVM) in snakes. Ancestral state reconstruction using maximum likelihood following the topology of Tonini et al. 2016 was employed in Mesquite. Author: Petra Frýdlová.

**SI 5**. Figure. Visualisation of the caudal part of the body and tail of the Kenyan sand boa (*Eryx colubrinus*) by µCT. (a) Cross-section view on the caudal part of the body around 2 cm anterior to the cloaca, (b) dorsal view on the caudal part of the body around 2 cm anterior to the cloaca and tail; (c) detailed dorsal view on the tip of the tail. The small, coloured structures are osteoderms. Colours are according to the volume (in mm^3^) of osteoderms. The scale of volume is similar for all views. Osteoderms do not cover the body continuously; rather they are individually distributed across the surface inside the skin. The distribution of osteoderms is regular resembling the distribution of scales. Bar 10 mm. Author: Jan Dudák and Petra Frýdlová.

**SI 6.** Figure. Visualisation of the caudal part of the body and tail of the rough-scaled sand boa (*Eryx conicus*) by µCT. (a) Cross-section view and (b) lateral view on the caudal part of the body around 2 cm anterior to the cloaca; (c) detailed lateral view on the tip of the tail. The small, coloured structures are osteoderms. Colours are according to the volume (in mm^3^) of osteoderms. The scale of volume is similar for all views. Osteoderms do not cover the body continuously; rather they are individually distributed across the surface inside the skin. The distribution of osteoderms is regular resembling the distribution of scales. Bar 10 mm. Author: Jan Dudák and Petra Frýdlová.

**Supplementary Video 1**. Video file. Visualization of osteoderms on the body of the adult rough-tailed sand boa (*Eryx conicus*) by µCT. The small colored structures are osteoderms, which are present on the tail and the caudal part of the body anterior to the cloaca. Colours are according to the volume of osteoderms (in mm^3^) going from the smallest violet, through shades of blue, green, and orange to the largest red ones (range: 9.74E-07 - 0.07 mm^3^). Osteoderms do not cover the body continuously; rather they are individually distributed across the surface inside the skin. The distribution of osteoderms is regular resembling the distribution of scales. Author: Jan Dudák.

**Supplementary Video 2**. Video file. Visualization of osteoderms on the body of the adult Kenyan sand boa (*Eryx colubrinus*) by µCT. The small colored structures are osteoderms, which are present on the tail and the caudal part of the body anterior to the cloaca. Colours are according to the volume of osteoderms (in mm^3^) going from the smallest violet, through shades of blue, green, and orange to the largest red ones (range: 9.93E-04 - 0.81 mm^3^). Osteoderms do not cover the body continuously; rather they are individually distributed across the surface inside the skin. The distribution of osteoderms is regular resembling the distribution of scales. Author: Jan Dudák.

**SI 7**. Figure. Visualisation of the caudal part of the body and tail of *Eryx miliaris* by µCT. (a) Lateral view on the caudal part of the body around 1 cm anterior and 1 cm behind the cloaca; (b) lateral view on the proximal part of the tail; (c) detail lateral view on the tip of the tail. The small, coloured structures are osteoderms. Colours are according to the volume (in mm^3^) of osteoderms. The scale of volume is similar for all views. Osteoderms do not cover the body continuously; rather they are individually distributed across the surface inside the skin. The distribution of osteoderms is regular resembling the distribution of scales. Bar 10 mm. Author: Jan Dudák and Petra Frýdlová.

**Supplementary Video 3**. Video file. The visualization of osteoderms on the body of the adult sand boa (*Eryx miliaris*) by µCT. The small colored structures are osteoderms, which are present on the tail and the caudal part of the body anterior to the cloaca. Colours are according to the volume of osteoderms (in mm^3^) going from the smallest violet, through shades of blue, green, and orange to the largest red ones (range: 1.46E-04 - 3.35E-02 mm^3^). Osteoderms do not cover the body continuously; rather they are individually distributed across the surface inside the skin. The distribution of osteoderms is regular resembling the distribution of scales. Author: Jan Dudák.

**SI 8**. Figure. Visualisation of the caudal part of the body and tail of *Eryx tataricus* by µCT. (a) Cross-section view on the caudal part of the body around 2 cm anterior to the cloaca, (b) lateral view on the middle part of the tail; (c) detailed lateral view on the tip of the tail. The small, coloured structures are osteoderms. Colours are according to the volume (in mm^3^) of osteoderms. The scale of volume is similar for all views. Osteoderms do not cover the body continuously; rather they are individually distributed across the surface inside the skin. The distribution of osteoderms is regular resembling the distribution of scales. Bar 10 mm. Author: Jan Dudák and Petra Frýdlová.

**Supplementary Video 4**. Video file. Visualization of osteoderms on the body of the adult sand boa (*Eryx tataricus*) by µCT. The small colored structures are osteoderms, which are present on the tail and the caudal part of the body anterior to the cloaca. Colours are according to the volume of osteoderms (in mm^3^) going from the smallest violet, through shades of blue, green, and orange to the largest red ones (range: 1.45E-04 - 0.37 mm^3^). Osteoderms do not cover the body continuously; rather they are individually distributed across the surface inside the skin. The distribution of osteoderms is regular resembling the distribution of scales. Author: Jan Dudák.

Table S1. Body size expressed as snout to vent length (SVL) separately for males and females of studied species in alphabetical order.

| **Species** | **SVL mean male (cm)** | **SVL mean female (cm)** | **References** |
| --- | --- | --- | --- |
| *Acrantophis madagascariensis* | 240* | 240* | 1 |
| *Boa imperator* | 116.40 | 147.80 | 2 |
| *Calabaria reinhardtii* | 77.60 | 77.60 | 3 |
| *Candoia aspera* | 42.79 | 58.80 | 4 |
| *Candoia carinata* | 46.02 | 66.13 | 5 |
| *Chilabothrus angulifer* | 160.14 | 180.88 | 6 |
| *Corallus hortulanus* | 123.30 | 138.90 | 7 |
| *Epicrates maurus* | 108 | 119 | 8 |
| *Eryx colubrinus* | 38.99 | 52.71 | 9 |
| *Eryx conicus* | 32.41 | 52.84 | 10 |
| *Eryx jaculus* | 35.50 | 42.60 | 11 |
| *Eryx johni* | 59.79 | 76.22 | 12 |
| *Eryx miliaris* | 30.70 | 30.60 | 13 |
| *Eryx muelleri* | 30.27 | 39.96 | 10 |
| *Eryx tataricus* | 32 | 32 | 14 |
| *Eunectes murinus* | 190.92 | 277.05 | 15 |
| *Gonionotophis poensis* | 108 | 108 | 16 |
| *Hemorrhois ravergieri* | 79.50 | 84.18 | 17 |
| *Lampropeltis ruthveni* | 127* | 127* | 18 |
| *Liasis mackloti* | 143.2** | 204.80** | 19 |
| *Lichanura trivirgata* | 55.50 | 61.40 | 20, 21 |
| *Python regius* | 111.30 | 116.20 | 22 |
| *Thamnophis sirtalis* | 45.30 | 51.50 | 23 |
| *Tropidophis melanurus* | 83 | 98.62 | 24 |
| *Tropidophis pardalis* | 22 | 30.40 | 25 |
| *Xenopeltis unicolor* | 52 | 52.30 | 26 |
| *Xerotyphlops vermicularis* | 20.49 | 20.49 | 27 |

**Footnote.** * Data were available only for total body length; ** computed from total body length (tail length is 15%).

**References.**

1. https://en.wikipedia.org/wiki/*Acrantophis_madagascariensis*

2. Boback, S. M. (2006). A morphometric comparison of island and mainland boas (*Boa constrictor*) in Belize. Copeia, 2006(2), 261-267.

3. Han, D., & Young, B. A. (2018). The rhinoceros among Serpents: Comparative anatomy and experimental biophysics of Calabar burrowing python (*Calabaria reinhardtii*) skin. Journal of Morphology, 279(1), 86-96.

4. Harlow, P., & Shine, R. (1992). Food habits and reproductive biology of the Pacific island boas (*Candoia*). Journal of Herpetology, 60-66.

5. McDowell, S. B. (1979). A catalogue of the snakes of New Guinea and the Solomons, with special reference to those in the Bernice P. Bishop Museum. Part III. Boinae and Acrochordoidea (Reptilia, Serpentes). Journal of Herpetology 13: 1 - 92.

6. Sheplan, B. R., & BR, S. (1974). Hispaniolan boas of the genus Epicrates (Serpentes, Boidae) and their Antillean relationships.

7. Pizzatto, L., & Marques, O. A. (2007). Reproductive ecology of boine snakes with emphasis on Brazilian species and a comparison to pythons. South American Journal of Herpetology, 2(2), 107-122.

8. Lourdais, O., Shine, R., Bonnet, X., & Brischoux, F. (2006). Sex differences in body composition, performance and behaviour in the Colombian rainbow boa (*Epicrates cenchria maurus*, Boidae). Journal of Zoology, 269(2), 175-182.

9. Lanza, B., & Nistri, A. (2005). Somali Boidae (genus Eryx Daudin 1803) and Pythonidae (genus Python Daudin 1803)(Reptilia Serpentes). Tropical Zoology, 18(1), 67-136.

10. Tokar, A. A. (1995). Taxonomic revision of the genus Gongylophis Wagler 1830: *G. conicus* (Schneider 1801) and *G. muelleri* Boulenger 1892 (Serpentes Boidae). Tropical Zoology, 8(2), 347-360.

11. Eskandarzadeh, N., Rastegar-Pouyani, N., Rastegar-Pouyani, E., Todehdehghan, F., & Rajabizadeh, M. (2018). Sexual Dimorphism in the Javelin Sand Boa, *Eryx jaculus* (Linnaeus, 1758)(Serpentes: Erycidae), from Western Iran. Current herpetology, 37(1), 88-92.

12. unpublished data Šimková & Frynta

13. Sheikh, Y., Orate, H., & Rastegar-Pouyani, E. (2019). Morphometric versus genomic evidence. The systematic status of *Eryx miliaris* (PALLAS, 1773), from Sistan, East Iran. HERPETOZOA, 31(3-4), 133-144.

14 Khan, M. S. (2002). A Guide to the Snakes of Pakistan (Vol. 16). Frankfurt am Main: Edition Chimaira.

15. Dirksen, L. (2002). Anakondas: monographische Revision der Gattung *Eunectes* Wagler, 1830 (Serpentes, Boidae). NTV.

16. Broadley, D. G., Tolley, K. A., Conradie, W., Wishart, S., Burger, M., ... & Greenbaum, E. (2018). A phylogeny and genus-level revision of the African file snakes *Gonionotophis* Boulenger (Squamata: Lamprophiidae). African Journal of Herpetology, 67(1), 43-60.

17. Moshtaghie, M., Kaboli, M., & Salehi, M. (2018). Geometric morphometric analysis of head shape in a spotted whip snake (*Hemorrhois ravergieri*) in different habitats of Iran. Acta Medica Mediterranea, 34, 583-589.

18. https://en.wikipedia.org/wiki/Lampropeltis_ruthveni

19. Carmichael, C. K. (2007). Phylogeography of the Indonesian water python, *Liasis mackloti* ssp.(Squamata: Boidae: Pythoninae): A comparative approach toward resolving phylogeny. The University of Southern Mississippi.

20. Klauber, L. M. (1933). Notes on *Lichanura*. Copeia, 1933(4), 214-215.

21. Kurfess, J. F. (1967). Mating, gestation, and growth rate in *Lichanura r. roseofusca*. Copeia, 1967(2), 477-479.

22 Aubret, F., Bonnet, X., Harris, M., & Maumelat, S. (2005). Sex differences in body size and ectoparasite load in the ball python, *Python regius*. Journal of Herpetology, 39(2), 315-320.

23. Shine, R. (1994). Sexual size dimorphism in snakes revisited. Copeia, 326-346.

24. Rodríguez-Cabrera, T. M., Saval, E. M., Navarro, R. A., Piggot, J. Q., Rodríguez-González, A. M., & Torres, J. (2021). Giant dwarfs: Very large giant tropes *Tropidophis melanurus* (Squamata: Tropidophiidae), and new maximum size records for the species. Reptiles & Amphibians, 28(3), 404-410.

25. Hedges, S. B., & Garrido, O. H. (1992). A new species of *Tropidophis* from Cuba (Serpentes: Tropidophiidae). Copeia, 820-825.

26. Leviton, Alan. (1983). Contributions to a review of Philippine snakes, XIV. The Snakes of the Genus *Xenopeltis, Zaocys, Psammodynastes*, and *Myersophis*. Philippine Journal of Science. 112. 195-223.

27. Afsar, M., Çiçek, K., Tayhan, Y., & Tok, C. V. (2016). New records of Eurasian Blind Snake, *Xerotyphlops vermicularis* (Merrem, 1820) from the Black Sea region of Turkey and its updated distribution. Biharean Biologist, 10(2), 98-103.
