# Supplementary figures and images for: The first description of dermal armour in snakes
Source: Sci Rep. 2023 Apr 19;13:6405. doi: 10.1038/s41598-023-33244-6 (PMC10115820; doi:10.1038/s41598-023-33244-6)

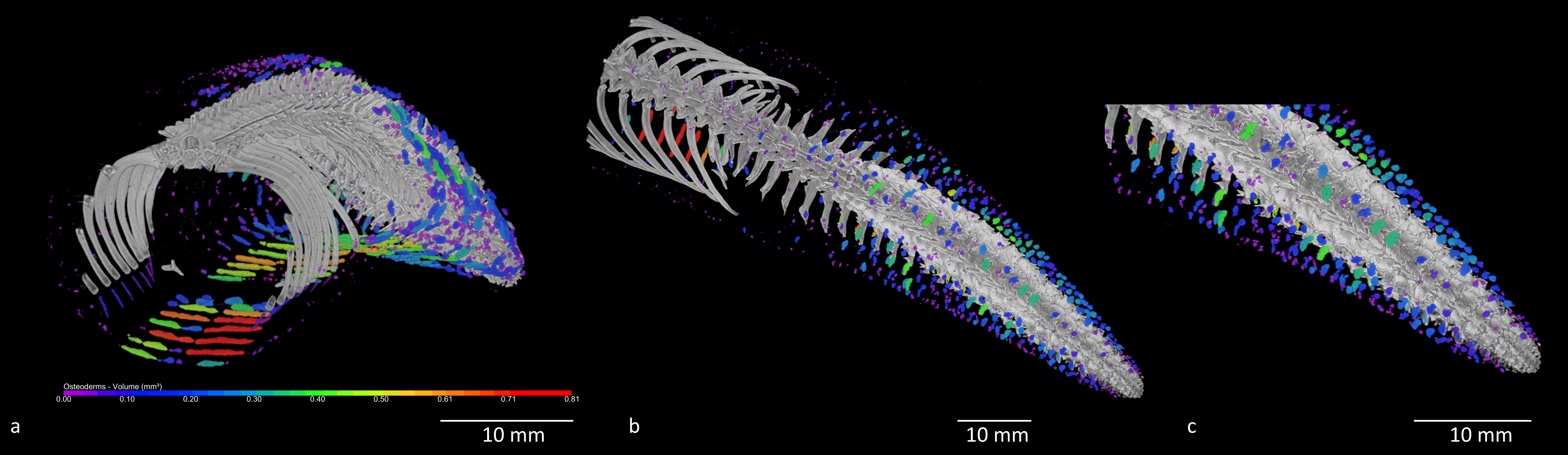

Supplement: Supplementary file 5 — Supplementary Information 5. [file 41598_2023_33244_MOESM5_ESM.tiff]

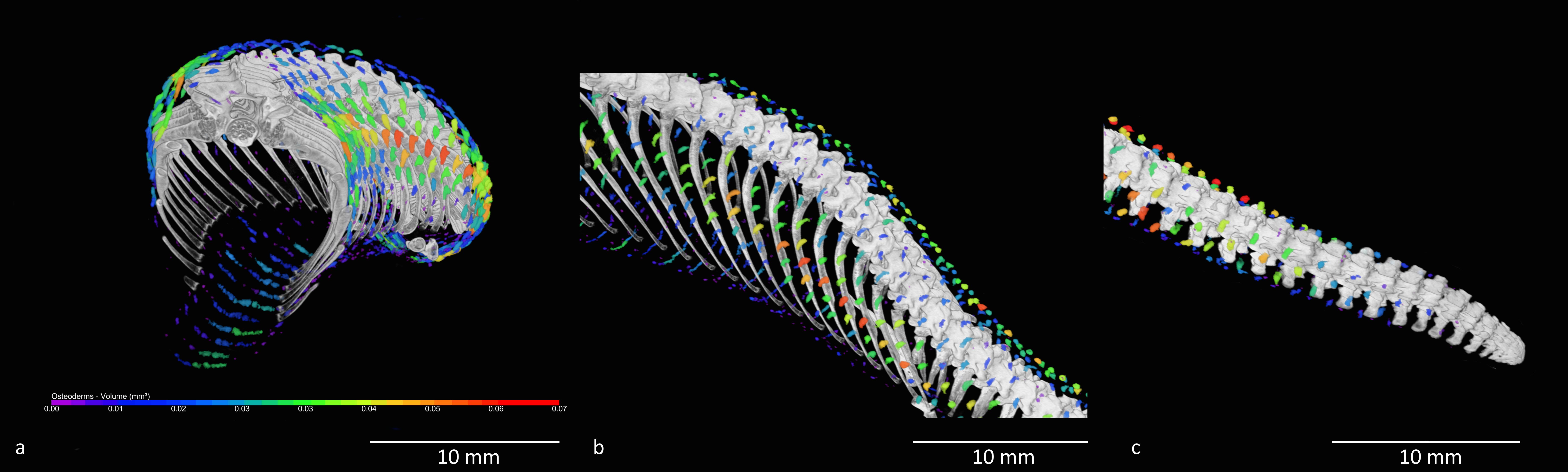

Supplement: Supplementary file 6 — Supplementary Information 6. [file 41598_2023_33244_MOESM6_ESM.tiff]

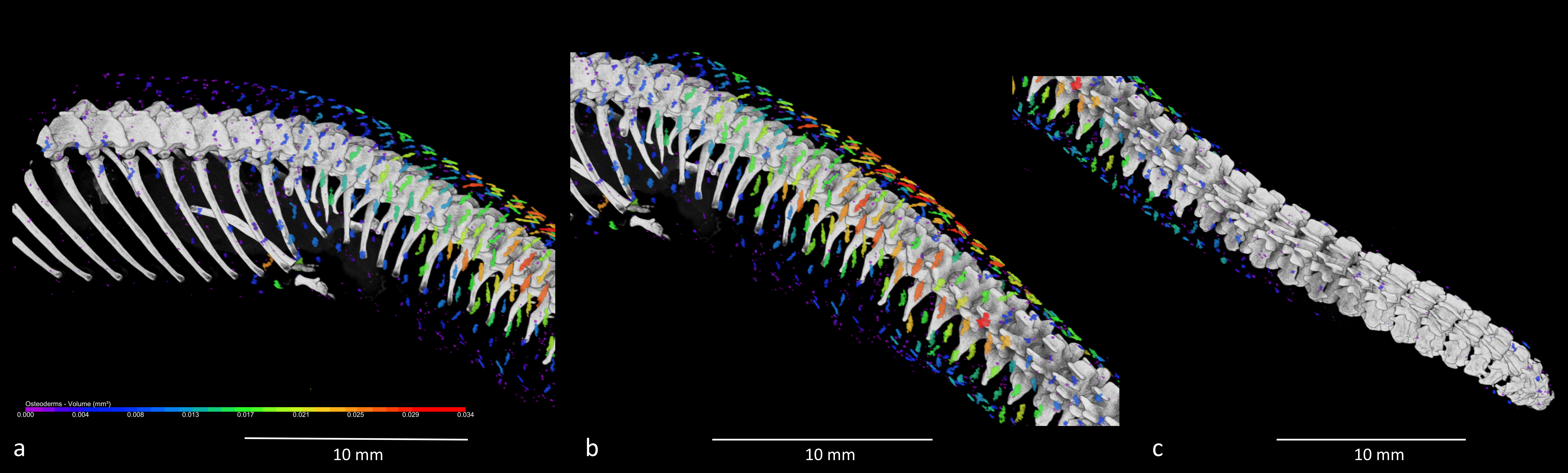

Supplement: Supplementary file 9 — Supplementary Information 7. [file 41598_2023_33244_MOESM9_ESM.tiff]

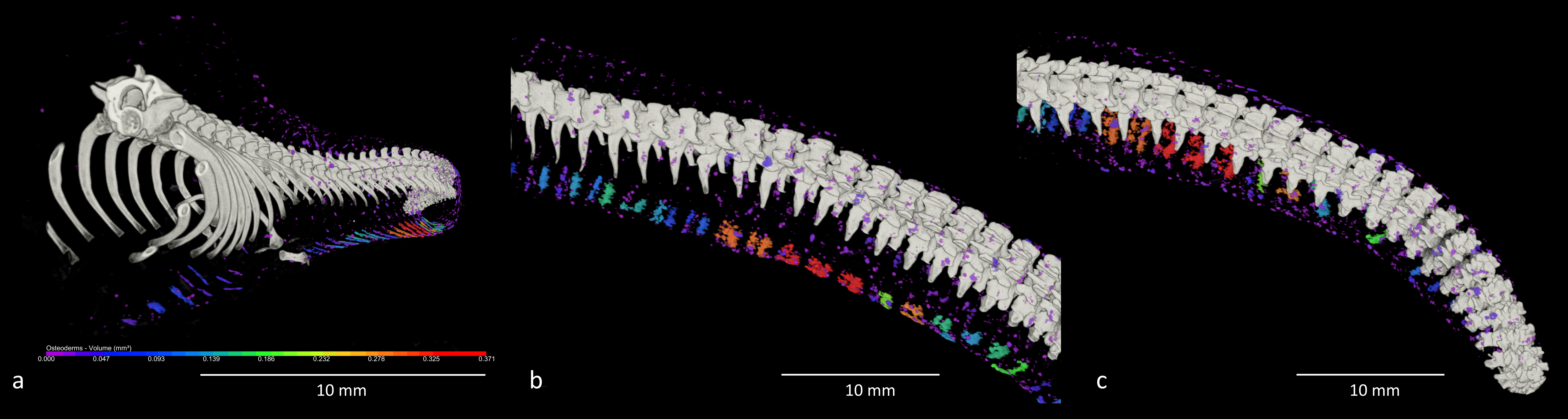

Supplement: Supplementary file 11 — Supplementary Information 8. [file 41598_2023_33244_MOESM11_ESM.tiff]
